# Emerging Applications of Intraoperative Optical Coherence Tomography in Corneal Surgery: A Narrative Review

**DOI:** 10.3390/jcm13185426

**Published:** 2024-09-13

**Authors:** Eleftherios Chatzimichail, Georgios Chondrozoumakis, Farideh Doroodgar, Efstathios Vounotrypidis, Georgios D. Panos, Zisis Gatzioufas

**Affiliations:** 1 Department of Ophthalmology, University Hospital of Basel, 4031 Basel, Switzerland; 2 Department of Ophthalmology, University Hospital of Heraklion, 71500 Heraklion, Greece; 3 Translational Ophthalmology Research Center, Tehran University of Medical Sciences, Tehran QF86+QC7, Iran; 4 Department of Ophthalmology, Ulm University, Prittwitzstraße 43, 89075 Ulm, Germany; 5 Department of Ophthalmology, School of Medicine, Aristotle University of Thessaloniki, 54124 Thessaloniki, Greece; 6 Division of Ophthalmology & Visual Sciences, School of Medicine, University of Nottingham, Nottingham NG7 2UH, UK

**Keywords:** intraoperative OCT, anterior segment OCT, corneal surgery, corneal transplantation

## Abstract

Intraoperative OCT (iOCT) is an innovative imaging modality that provides ophthalmic surgeons with real-time cross-sectional views of the surgical field. Recent advances in OCT technology, such as higher acquisition scanning rates, enable real-time video visualization. iOCT systems are widely used in both vitreoretinal and anterior segment surgeries. In corneal surgeries, iOCT imaging aims to optimize efficacy and safety by improving depth perception and enhancing visualization in cases of opaque optical media. iOCT is a valuable tool not only for experienced corneal surgeons, but also for training novice surgeons. This review summarizes the emerging applications of iOCT in corneal surgery, particularly in technically demanding lamellar keratoplasty procedures, as well as in various other corneal diseases and complications that require surgical intervention.

## 1. Introduction

In 1994, Izatt et al. demonstrated for the first time the anterior segment optical coherence tomography (AS- OCT), using light with an 830 nm wavelength [1]. AS-OCT offers the ability to assess the structures of anterior segments like the cornea, conjunctiva, sclera, rectus muscles, anterior chamber angle structures, and lens [2]. Of great importance is the noninvasive nature of OCT, as well as the ease of using the device and direct interpretation of the images by physicians, even those without much experience [3].

AS OCT provides static images and cannot be used to perform real-time assessments. Until recently, the assessment of ocular tissues and the interaction of tissues and instruments intraoperatively was based on visualization through an operating microscope en face [4]. Intraoperative OCT (iOCT), an emerging imaging modality, is has become more favored lately as it allows for real-time cross-sectional imaging during surgery. The first use of iOCT was reported in anterior segment surgery performed in 2005 by Geerling et al., who performed a lamellar keratoplasty and trabeculectomy using an OCT with a wavelength of 1310 nm [5]. Subsequently, broad use of iOCT has been reported in the literature including in vitreoretinal surgery, for indications such as retinal detachment, epiretinal membrane and macular holes, among others [6,7,8,9,10,11,12,13,14].

The usefulness of iOCT in corneal surgery has been well documented in many studies, like PIONEER and DISCOVER, including, among others, full thickness keratoplasty and lamellar keratoplasty procedures such as deep anterior lamellar keratoplasty (DALK), Descemet stripping automated endothelial keratoplasty (DSAEK) and Descemet membrane endothelial keratoplasty (DMEK) [15,16,17]. This technology can be extremely useful particularly in challenging cases with poor visualization due to corneal scarring or decompensation, providing visualization of the structures underneath the cornea, which otherwise are difficult to recognize with an operating microscope [4]. 

For this mini review, a search across PubMed, Embase and Scopus using the keywords “(intraoperative OCT* OR Optical Coherence Tomography)”, “(intraoperative OCT* OR Optical Coherence Tomography) AND (cornea OR corneal surgery)”, “(intraoperative OCT* OR Optical Coherence Tomography) AND (corneal transplantation)” was conducted. We included the most meaningful studies. Studies published in languages other than English were excluded.

In this context, the primary aim of this narrative review is to highlight the applications of iOCT in modern corneal surgery including both penetrating and lamellar procedures, emphasizing also on the advantages of this revolutionary technology.

## 2. Optical Coherence Tomography—Principles and Technology

OCT is a modality that uses low coherence interferometry principles to produce in vivo imaging of ocular structures, in a noninvasive manner. This technology is widely used in the clinical evaluation of ocular conditions of both the anterior segment (AS) and retina [18,19]. It offers higher resolution images than ultrasound tomography and a higher depth of penetration than confocal microscope [20,21].

Time domain (TD) OCT and Fourier domain (FD) OCT are the two main methods of implementation. TD-OCT utilizes a moving reference arm to obtain a high depth resolution. Interference occurs only when the reference distance length is matched to the distance of each scattering point in a tissue in an A-scan [18]. The movement delay is the major limitation of TD-OCT [18].

FD-OCT produces spectrally encoded interference patterns for each A-scan at once, thus enabling much higher acquisition rates. FD-OCT is further divided into spatially encoded Fourier-domain OCT, often called spectral-domain OCT(SD-OCT) and time encoded Fourier domain OCT, often called swept-source OCT (SS-OCT). The first modality achieves interference with a broadband light source and dispersive detector and the latter with a tunable narrowband light source and a point detector. Both FD-OCT scanners achieve higher scan speeds (SS-OCT up to 100,000/s) and better axial resolutions (SD-OCT up to 5 μm) than TD-OCT [22].

On the other hand, TD-OCT offers superior scan depths and scan widths in comparison to FD-OCT systems. However, many of the modulations available for FD systems match these characteristics, especially in terms of scan width [22].

The wavelength of choice for AS-OCT devices is 1300 nm, providing the advantage of deeper tissue penetration, which is required for the visualization of angle structures. Although water absorption at this wavelength is generally higher, in retinal OCT, the aqueous component of the vitreous humor does not interfere with the target tissues, ensuring that image quality remains unaffected. On the contrary the vitreous body constitutes a protective barrier for the retina, allowing even higher light power settings to be utilized [22].

iOCT on the other hand is aimed towards real-time image construction, allowing for it to be used intraoperatively [23]. This is achieved through a high-power broadband source which is required to provide adequate illumination in a short amount of time, real-time image acquisition hardware and a high-speed scanning delay line in the reference arm based on Fourier-transform pulse shaping technology [24]. A-scans must be collected at a rate equal to the frame rate (images per second) times the number of A-scans per frame. For instance, in order for four images to be acquired per second with 250 lines (A-scans) per image, the delay line must scan at 1000 scans per second. In order to capture images at video rate (30 frames per second) with 100 lines per frame, 3000 scans per second are required [24].

With the recent introduction of real-time intraoperative iOCT integrated into a microscope, real-time imaging of the tissues of surgical interest and tissue–instrument interactions have become feasible. The integration of iOCT into microscopes through external mounts (MI-OCT) provides increased stability, along with improved image reproducibility and foot pedal control for the X-Y-Z translation [16]. In 2016, a swept source four-dimensional microscope-integrated optical coherence tomography was introduced which is capable of processing OCT volumes at up to 10 volumes per second, allowing for the imaging ocular structures that could not be seen through a surgical microscope [25].

To date, there are three commercially available MIOCT systems that we are aware of: Rescan^®^ 700 (Cirrus OCT system built on Lumera 700 microscope; Carl Zeiss Meditec, Oberkochen, Germany) [26], OPMedT^®^ (OPMedT OCT system, HaagStreit Hi-R 1000G-microscope, Haag-Streit Surgical GmbH, Wedel, Germany) [27], and EnFocus^®^ (Bioptigen, Leica, Wetzlar, Germany) [9,28,29].

## 3. iOCT in Corneal Surgery

Although previously the main indications for the use of iOCT have been lamellar corneal transplantation procedures, such as DALK and DMEK, a variety of applications have emerged in other conditions as well, including corneal trauma, the management of descemetolysis and intracorneal ring segment implantation [15]. 

## 4. Penetrating Keratoplasty

For over a century, the penetrating keratoplasty (PK) has been the surgical method of choice for the treatment of various corneal diseases [30]. Eguchi et al. mentioned the importance of iOCT in corneal suturing during PK, allowing for the visualization of the needle penetrating the cornea layers as well as enabling the surgeon to adjust the sutures to achieve proper alignment, thus preventing the manifestation of significant surgically induced astigmatic effects [31,32]. In addition, iOCT offers continuous evaluation of the graft–host interface and it is a useful tool for detecting possible wound dehiscence [15]. In complex cases with anterior segment malformations, iOCT provides visualization of the iris and angle structures, revealing potential iridocorneal adhesions which are otherwise difficult to identify with a microscope, due to corneal opacification [26]. An assessment of the host’s cornea trephination depth can also be facilitated by iOCT in challenging anterior segment conditions, avoiding iris trauma [33]. In terms of pediatric keratoplasty, Sharma et al., in a comparative study, reported significantly fewer secondary interventions in the group in which the operation was carried out with the aid of iOCT [34].

## 5. Deep Anterior Lamellar Keratoplasty

Over the last 20 years there has been a gradual shift from PK to lamellar corneal transplantation procedures [35]. The deep anterior lamellar keratoplasty (DALK) is the corneal transplantation method of choice in advanced keratoconus, preserving host corneal endothelium and thereby resulting in reduced risk of corneal graft rejection [36]. However, the steep learning curve involved represents the main drawback of this technically demanding surgical procedure. Intraoperative OCT can offer important assistance in almost all steps of DALK and prevent potential complications, thus improving surgical outcomes.

**Figure 1:**
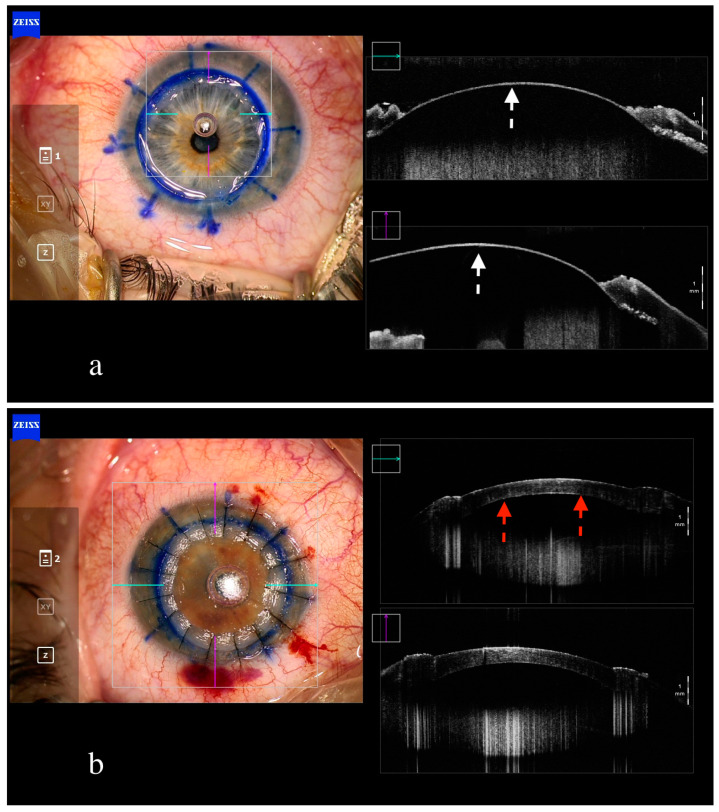
Intraoperative anterior segment OCT in big bubble DALK. Optimal separation of Descemet membrane ((**a**), white arrows). Complete attachment of Descemet membrane (red arrows) at the end of surgery, without formation of double chamber (**b**).

To begin with, iOCT improves depth perception, which is hindered by the en face surgical microscope view, enhancing the efficiency of partial corneal preparation, manual stromal dissection and canula/needle insertion in the big bubble technique, as shown in the PIONEER study (Figure 1a) [37]. In particular, the depth of trepanation can be accurately estimated, avoiding corneal perforation [37,38].

Particularly in the big bubble technique, the injection of air into the posterior stroma can be visualized through iOCT, as hyperacoustic signaling [39]. The formation of a big bubble as well as the presence of a ‘double’ anterior chamber at the end of the surgery can be evaluated with the aid of iOCT (Figure 1b). Finally, iOCT offers the same advantages to this procedure as in PK, particularly regarding suturing and tissue apposition alignment [37].

## 6. Endothelial Keratoplasty

Regarding endothelial keratoplasty, in 2015, Saad et al. first described the application of iOCT in DMEK in a prospective case series of 14 patients, and reported excellent outcomes even in complex cases [40].

**Figure 2:**
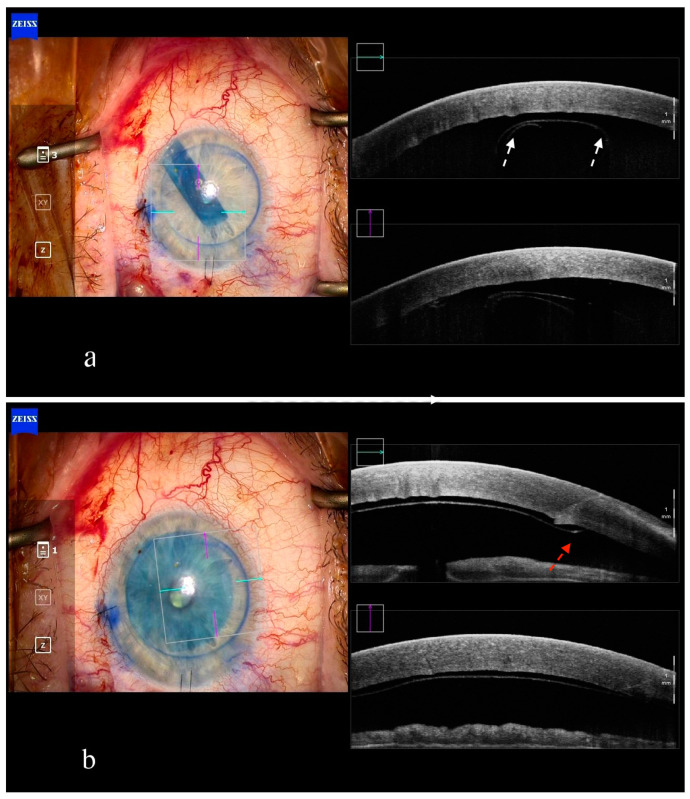
Intraoperative anterior segment OCT in DMEK. Visualization of the graft scrolling ((**a**), white arrows) and the graft edge ((**b**), red arrow) is valuable for defining the graft orientation.

The use of iOCT in DMEK is helpful due to the enhanced visualization of rolling, unfolding, the orientation of the graft and, finally, the attachment of the graft to the cornea of the recipient (Figure 2) [27]. Peripheral anterior synechiae can also be identified when present intraoperatively [41]. The assessment of graft orientation with iOCT is essential in eyes with excessive corneal edema or haze as well as in cases in which no graft orientation markings are used [31].

Another implication of iOCT in DMEK is the assistance it can provide in descemetorhexis in situations with poor visualization due to an opaque or edematous cornea [42]. Thus, the initial results from the DISCOVER study demonstrate an overall reduction in the complication rate and intraoperative time spent for unscrolling the graft with the use of iOCT [43].

**Figure 3:**
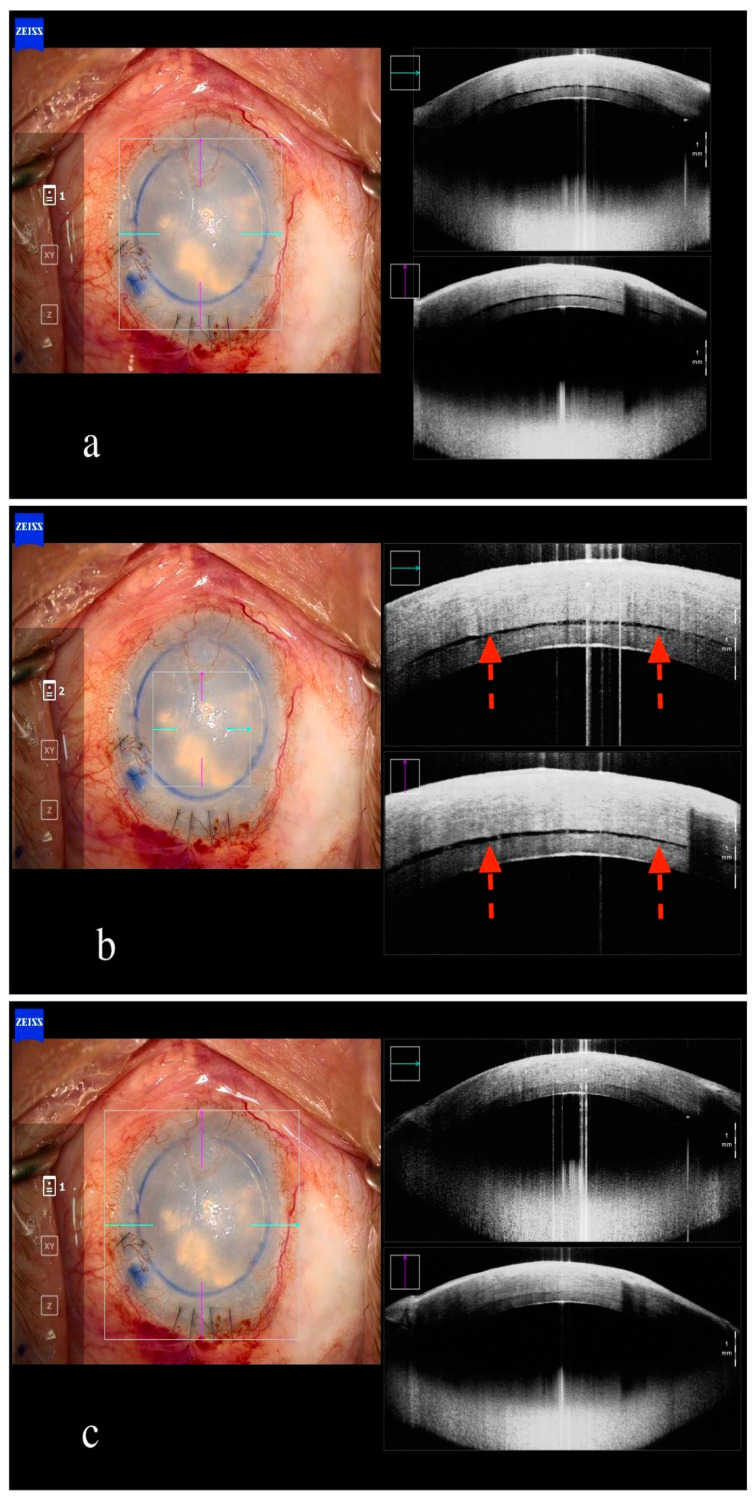
Intraoperative anterior segment OCT in ultrathin DSAEK. Residual fluid in recipient cornea-graft interface ((**a**,**b**), red arrows). Residual fluid fully resolved after massage of recipient cornea (**c**).

Regarding DSAEK, the presence residual interface fluid can be confirmed with iOCT and adequately addressed, as demonstrated by the results of the PIONEER and DISCOVER studies (Figure 3) [16,17].

## 7. Other Corneal Surgeries

**Figure 4:**
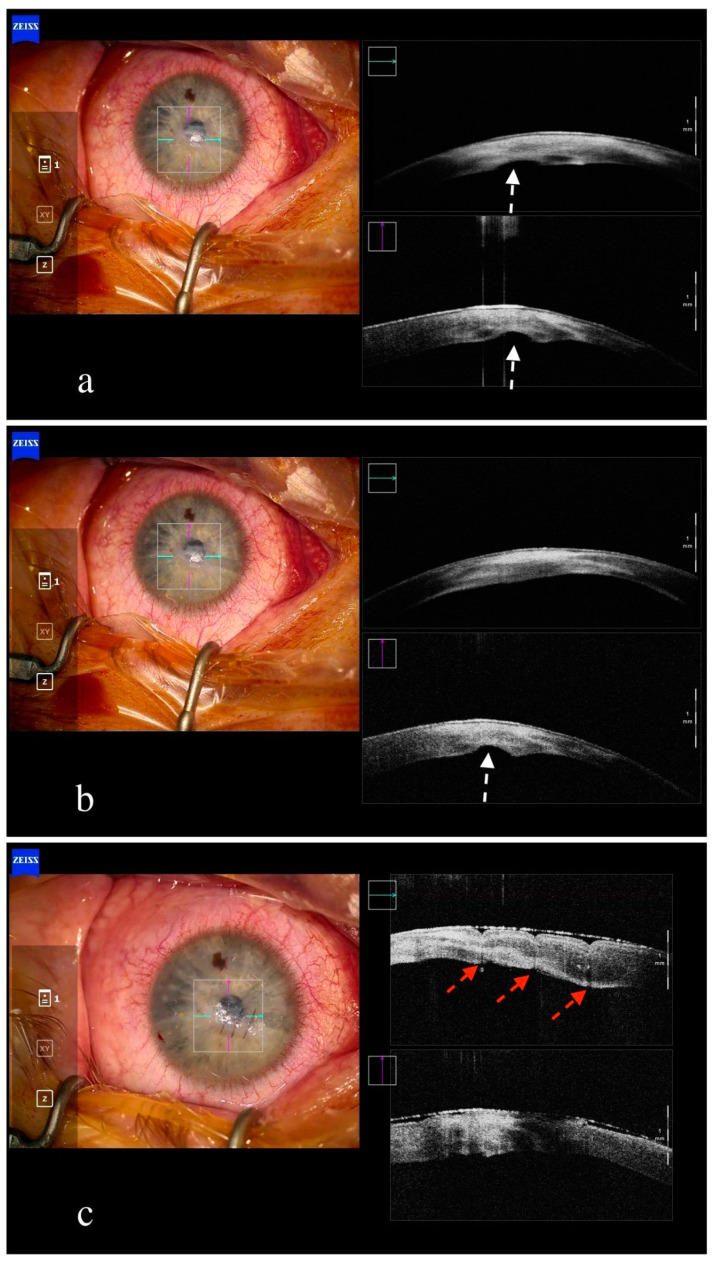
Intraoperative anterior segment OCT in acute corneal hydrops treated with compression pre-descemetic sutures (‘Murraine sutures’). Break in Descemet membrane is visualized in acute corneal hydrops ((**a**,**b**), white arrows). Closure of Descemet membrane break after placement of Murraine sutures ((**c**), red arrows).

In addition to corneal transplantation, iOCT has also been proved to be helpful in a variety of corneal conditions requiring surgical intervention. In corneal trauma, iOCT can determine the depth of corneal laceration and facilitate the removal of potential foreign bodies, decreasing the risk of complications [30]. Furthermore, adequate suturing can be accomplished under iOCT guidance in complex cases of corneal laceration [4]. Moreover, Descemet membrane breaks can be identified with the aid of iOCT in cases of acute corneal hydrops and proper suturing with pre-descemetic compression sutures (Muraine sutures) can be successfully achieved (Figure 4) [44]. Finally, our group has published the successful application of iOCT in challenging corneal cases, such as in DSAEK-tamponade for spontaneous corneal perforation, as well as in the removal of misdirected intracorneal viscoelastic substances [45,46].

Ocular surface diseases like pterygium, squamous surface neoplasia, corneal dermoid and Salzman corneal degeneration can be successfully managed with the aid of iOCT which serves as a guide for complete removal [33]. In particular, Ehlers et al., in the DISCOVER study, refer to the utilization of iOCT for the visualization and recognition of the pathologically healthy cornea cleavage plane in Salzman corneal degeneration [17].

In the same study, the insertion of intracorneal ring segments was navigated with iOCT for accurate corneal incision and channel creation [17]. Retrocorneal, post-PK fibrous membrane excision is another iOCT-assisted procedure described by Ruland et al. [47]. Furthermore, a case of iOCT-guided corneal biopsy has been reported by Schmidt et al., indicating another potential application [48]. Excimer laser-assisted phototherapeutic keratectomy (PTK) for corneal scars could also be aided by iOCT technology, as reported by Siebleman et al. [49].

Further applications include the management of descemetolysis with SF6 gas-tamponade and also the creation of an intrastromal pocket for Bowman layer transplantation in patients with keratoconus, where iOCT can provide maximal precision and safety [50].

## 8. Conclusions

Microscope-integrated optical coherence tomography has revolutionized corneal surgery providing precise real-time visualization of the corneal tissue. One of the fields that has advanced rapidly through the introduction of iOCT is corneal transplant surgery. The major contribution of iOCT to corneal transplantation is the important addition of depth perception to the en face microscope view of the surgeon, maximizing the efficiency and safety of various procedures. This advantage could be valuable particularly for inexperienced surgeons and, at the same time, it could assist novice surgeons in terms of training.

Further improvements in iOCT technology are slowly emerging. In future platforms, swept source OCT will be an important upgrade, aiming to address some of the current limitations of integrated OCT systems. The increased acquisition speed with high rates of A-scans will enable the visualization of real-time 3D instrument–tissue interactions. This volumetric scanning at near video rates is possible given exceptional speed of the swept source system. Software additions can also be enhanced to provide surgeons automated tracking in the x-y-z axis [51].

However, the primary obstacle for the use of iOCT in every clinical practice is the high acquisition cost. However, it is expected that the cost of iOCT will decline significantly in the near future.

In conclusion, the introduction of iOCT to the operating theater is potentially a game changer in corneal surgery, assisting intraoperative decision making by visualizing the corneal tissue–surgical instruments interactions in real time. At the same time, it provides maximal safety by reducing complications, and increases efficiency by decreasing surgical time. Looking into the future, further development of the iOCT technology will possibly increase the impact on corneal surgery, resulting in improved clinical outcomes and a higher quality of life for patients undergoing corneal surgical procedures.

## Abbreviations

OCT	optical coherence tomography
DALK	deep anterior lamellar keratoplasty
DMEK	Descemet membrane endothelial keratoplasty
DSAEK	Descemet’s stripping endothelial keratoplasty
PK	penetrating keratoplasty
DM	Descemet membrane
